# Chemical Composition and Antioxidant Activity of the Stembark Essential Oils of Two *Cannabis sativa* L. Cultivars from Komga, South Africa

**DOI:** 10.3390/ijms26178552

**Published:** 2025-09-03

**Authors:** Anwuli E. Odieka, Ayodeji O. Oriola, Gugulethu M. Miya, Pallab Kar, Opeoluwa O. Oyedeji, Mavuto Gondwe, Yiseyon S. Hosu, Thami Madliwa, Adebola O. Oyedeji

**Affiliations:** 1 Department of Chemical and Physical Sciences, Walter Sisulu University, Mthatha 5099, South Africa; aoriola@wsu.ac.za (A.O.O.);; 2 African Medicinal Flora and Fauna Research Niche Area, Walter Sisulu University, Mthatha 5099, South Africa; pallabkar.bio@gmail.com; 3 Department of Chemistry, University of Fort Hare, Alice 5700, South Africa; 4 Department of Human Biology, Walter Sisulu University, Mthatha 5099, South Africa; 5 Department of Business Management and Economics, Walter Sisulu University, Mthatha 5099, South Africa; 6 Eastern Cape Hemp Producers Association (ECHPA), Komga 4950, South Africa

**Keywords:** *Cannabis sativa*, essential oils, gas chromatography-mass spectrometry, antioxidant activity, cannabinoids, molecular docking

## Abstract

*Cannabis sativa* L. is an aromatic medicinal plant with various biologically active classes of compounds such as cannabinoids, polyphenols, and terpenes. Unlike the widely investigated inflorescence and leaf, the stembark of *C. sativa* has been overlooked regarding its medicinal potential. This study, therefore, was aimed at determining the chemical composition and antioxidant activity of the essential oils (EOs) obtained from the fresh and dried stembark of two *C. sativa* cultivars, Lifter and Cherrywine, grown in Komga, South Africa, with a view to ascertaining the more promising cultivar. The chemical profiles of the hydro-distilled EOs were analyzed by gas chromatography-mass spectrometry (GC-MS), while an in vitro antioxidant activity assessment of the EOs was performed using DPPH and H_2_O_2_ spectrophotometric methods. The identified constituents from the EOs were molecularly docked against NOX2, a protein implicated in oxidative stress. The afforded EOs were colorless with a mild skunk-like odor. A total of thirty-two constituents were identified in both fresh and dry oils from the Lifter cultivar while the Cherrywine cultivar contained a total of forty-two constituents. The EOs of both cultivars contained twenty compounds, notably Cannabidiol (0.25–85.03%), Caryophyllene oxide (1.27–19.58%), Caryophyllene (0.64–16.61%), Humulene (0.37–8.15%), Octacosane (3.37–6.55%), Humulene-1,2-epoxide (0.45–5.78%), Nerolidol (0.32–4.99%), Palmitic acid (1.45–4.45%), Tetracosane (1.75–2.91%), Dronabinol (0.86–2.86%), Cannabinol (0.54–1.64%), 7-epi-γ-eudesmol (0.53–1.00%), Guaiol (0.37–0.66%), Linoleic acid (0.22–0.60%), γ-Selinene (0.15–0.48%), β-Eudesmol (0.34–0.50%), and Linalool (0.24–0.30%). The dried Lifter stembark oil (DLSO) gave the best antioxidant activity among the four investigated cannabis oils, exhibiting the lowest IC_50_ values of 21.68 ± 1.71 and 26.20 ± 1.34 µg/mL against DPPH and H_2_O_2_ radicals, respectively. The notable antioxidant activity of the DLSO may be attributed to the higher number (30) of constituents compared to the fresh Lifter stembark oil (LSO) with 11 constituents. Additionally, the DLSO showed a unique chemical profile comprising monoterpenes, oxygenated and hydrocarbon sesquiterpenes. Further in silico studies on the putative constituents in the Lifter cultivar revealed Cannabinol, Cannabidiol, and Linalool as the promising constituents based on their higher binding energy scores of −9.7, −8.5, and −6.5 kcal/mol, respectively, compared to L-Ascorbic acid (−5.7 kcal/mol). It can be inferred from this study that the EOs from the stembark of *C. sativa* contain promising compounds, such as Cannabinol, Cannabidiol, and Linalool, which might be responsible for the displayed antioxidant activity of the oils. Thus, the study findings underscore the biological importance of *C. sativa* stembark in the management of oxidative stress-related conditions.

## 1. Introduction

*Cannabis sativa* L., commonly referred to as “cannabis”, is an annual herb that belongs to the family Cannabaceae [1]. *C. sativa* is an example of a multi-purpose plant and contains a wide range of bioactive chemicals, including cannabinoids, phenolics, terpenes, flavonoids, essential oils, and alkaloids [2]. The plant’s leaves, bracts, and stems are rich in trichomes, which are a varied set of structures comprising secondary metabolites (phyto-cannabinoids and terpenoids) that are important for defense, plant interactions, and the characteristic scent [3]. However, its use is restricted in many areas, as it is only legal in a few countries due to its psychoactive nature [4]. The cellulosic fibers (bast fibers) are utilized to make bioplastics, a fiberglass alternative, while the woody fibers are used for animal bedding, functionalized fabrics, and anti-bacterial finishing agents [2]. Nevertheless, Cannabidiol (CBD), a notable phyto-cannabinoid, maintains a good safety profile [5]. Some of the therapeutic benefits of CBD oils when used alone or co-administered with Tetrahydrocannabinol (THC) include arthritis and joint pain, inflammation, depression, anxiety, cancer, cardiovascular diseases, multiple sclerosis, chronic pain, epilepsy, and seizure disorders [5,6,7,8,9,10]. Terpenoids are the second most abundant family of Cannabis phytochemicals, and they are responsible for their distinct scent [11]. One distinction of the plant *C. sativa* is its fragrance.

Essential oils (EOs) are volatile oily plant extracts derived through distillation (dry, steam, or hydro) or by mechanical methods without heating [12,13]. Essential oils contain terpenes, which give plants their distinct scent [14]. The output of essential oil from plants varies greatly, and the range is often between 0.05 and 18.0% [14]. According to Sankarikutty and Narayanan, the price of any given commercial grade of EO depends on the production rate, availability (percentage oil yield from the plant species), and applications [14]. The more volatile monoterpenes and sesquiterpenes are what give cannabis its aroma rather than the terpene-phenolic cannabinoids [15]. Cannabis terpenoids and flavonoids, specifically myrcene, limonene, pinene, caryophyllene, and cannflavin A, work synergistically with cannabinoids to produce pharmacological effects [16]. Cannabis essential oil has a wide range of known applications in aromatherapy, cosmetics, soaps, shampoos, creams, oils, fragrances, and food items, all of which have a typical cannabis scent [15]. Cannabis stems may contain phytochemicals of medicinal interest since quantities above 0.05% are pharmaceutically intriguing [17].

In the food industry, natural antioxidants play an important role in maintaining the normal growth of cells and tissues and protecting the human body against free radicals that could be generated by stress, ultraviolet rays, and vigorous workouts [18,19,20,21]. Reactive oxygen species (ROS) circulate freely with access to all organs and tissues, thereby causing tissue damage through various mechanisms. Thus, there is a need for continuous discovery of new natural antioxidants, which are known as compounds of different chemical nature that can eliminate or retard the free radical oxidation of organic compounds [18,22,23,24]. γ-Terpinene, α-Terpinene, β-Caryophyllene, Guaiol, and Terpinolene have been reported to have antioxidant activities [25]. As earlier stated, cannabis stems are currently being used as a source of fiber, livestock feeds, and biofuel, etc. [2]. There is limited literature on the medicinal potential of *C. sativa* stem bark, which is typically regarded as a waste material apart from its use as fibers [17,26,27]. Consequently, these insights provide a rationale for repurposing cannabis stem bark into useful resources for biological applications. This study primarily focused on assessing the chemical profiles of two commercially available cultivars and their antioxidant activities. Therefore, this study determined the chemical composition of the EOs from fresh and dried stembark of *C. sativa* using gas chromatography-mass spectrometry (GC-MS). The reliable and commonly used spectrophotometric (2,2-diphenyl-1-picrylhydrazyl (DPPH)) and hydrogen peroxide (H_2_O_2_) assays were adopted in this study to measure their radical scavenging activity. Furthermore, in silico studies to reveal the promising constituents based on their higher binding energy scores were performed.

## 2. Results and Discussion

### 2.1. Physicochemical Analysis of Fresh and Dried Stembark EOs from Two C. sativa Cultivars

The results are shown in Table 1. Upon hydro-distillation, the fresh Lifter stembark oil (LSO) and the dried Lifter stembark oil (DLSO) afforded yields of 0.53% and 0.67% (*w*/*w*), respectively. Conversely, the fresh and dried stembark oils of the Cherrywine cultivar (CSO and DCSO) had yields of 0.18% and 1.58% (*w*/*w*), respectively. Overall, the DCSO exhibited a higher oil yield compared to the DLSO, LSO, and CSO. However, the yield range for the fresh and dried stembark oils was found to be 0.05–18.0% (*w*/*w*), which aligns with the literature [14]. This study demonstrates that extracting essential oils (EOs) from dry *C. sativa* stem bark results in a higher oil yield. However, our findings contradict those of Ascrizzi et al., who indicated that hydro-distilled fresh materials yield more oil [28]. This suggests that the quality and quantity of EOs may depend on various factors such as chemotype, biotype, environmental or geographical cultivation, and climatic conditions [29]. The EOs obtained were colorless (transparent) and had a mildly pungent, skunk-like smell, which are typical characteristics of *C. sativa* oils. This could indicate oils with high sesquiterpene concentrations [15]. Sesquiterpenes such as caryophyllene, humulene, and others commonly found in cannabis are associated with skunky odors. Additionally, sesquiterpenes are more volatile than monoterpenes and tend to dominate in oils that possess less traditional floral fragrances [30].

### 2.2. Chemical Composition of Stembark EOs of Two C. sativa Cultivars

The terpenes and cannabinoids examined in this study are believed to encompass a variety of terpenes and cannabinoids detected in significant amounts in two cultivars of *C. sativa* sourced from South Africa. The notable quantitative and qualitative differences in the chemical compositions of EOs from the two cultivars, as revealed by the chromatographic profiles, are presented in Figures S1–S4 and Table 2. The dried plant materials (DLSO and DCSO) contained more chemical components. However, some EO constituents in the fresh plant part were absent after drying. Interestingly, this study suggests that drying the plant material did not cause a significant reduction in the chemical components. The main chemical constituents consistent in the stembark EOs of both cultivars include Caryophyllene oxide (1.27–19.58%), Caryophyllene (0.64–16.61%), Humulene (0.37–8.15%), Humulene -1,2-epoxide (0.45–5.78%), γ-Selinene (0.15–0.48%), Nerolidol (0.32–4.99%), Guaiol (0.37–0.66%), Ledol (0.18–0.21%), 7-epi-γ-eudesmol (0.53–1.00%), β-Eudesmol (0.34–0.50%), Palmitic acid (1.45–4.45%), Cannabidiol (0.25–85.03%), Dronabinol (0.86–2.86%), Cannabinol (0.54–1.64%), Octacosane (3.37–6.55%), Tetracosane (1.75–2.91%), Hexahydrofarnesyl acetone (0.27–0.40%), Linalool (0.24–0.30%), Ledol (0.18–0.21%) and Pentadecanoic acid (0.16–0.28%). The stembark oils contained predominantly oxygenated sesquiterpenes. The sesquiterpene found exclusively in the LSO is α-Eudesmol (0.39%).

Linalool and Terpineol were the monoterpenes present in the dried Lifter oil (DLSO), whereas sesquiterpenes such as Caryophyllene, Humulene, (+/−)-β-Cadinene, -γ-Selinene, Nerolidol, Isoaromadendrene epoxide, Bulnesol, and 5-epi-7-epi-α-Eudesmol were only present in the dried stembark oil in significant amounts. Caryophyllene has anti-inflammatory, analgesic, and antipyretic properties [31]. It is also used as a food flavoring agent. Humulene is a monocyclic sesquiterpene with anti-inflammatory and anticancer properties [32,33]. On the other hand, β-cis-Ocimene (0.33%) was the only monoterpene found exclusively in the fresh Cherrywine oil (CSO), while sesquiterpenes such as cis-α-Bergamotene, trans-α-Bisabolene epoxide and Caryophylla-4(12),8(13)-dien-5-β-ol, were only present in CSO. The monoterpenes found exclusively in the dried Cherrywine oil (DCSO) are α-Pinene (0.16%) and Fenchol (0.13%). Interestingly, some of the sesquiterpenes found exclusively in the DCSO, such as Zingiberenol, α-Calacorene, Caryophylla-4(12),8(13)-Dien-5-α-ol, α-Cubebene, α-Curcumene, and Bicyclo-[7.2.0]undecan-3-ol, 11,11-dimethyl-4,8-bis(methylene), have not been previously reported in *C. sativa.* Therefore, our study findings mark the first report of the aforementioned sesquiterpenes in the dried Cherrywine cultivar of *C. sativa* grown in Komga, South Africa. However, these sesquiterpenes have been found in other medicinal plants, with their biological activities reported. For example, Zingiberenol and α-Curcumene are sesquiterpenes present in Ginger and *Zingiberaceae* oils, reported to have antimicrobial, antioxidant, anti-cancer, and anti-inflammatory activities [34,35]. Similarly, Caryophylla-4(12),8(13)-Dien-5-α-ol and Caryophylla-4(12),8(13)-dien-5-β-ol found in CSO have been reported in *Psidium* species, with cytotoxic, antifungal, anti-inflammatory, larvicidal, antimicrobial and antioxidant properties [36,37]. It is worth mentioning that Caryophyllene, Caryophyllene oxide, and Humulene were present in both fresh and dried Cherrywine stembark oils in significant amounts and could serve as biomarkers for this cultivar.

Additionally, neutral cannabinoids were detected in the EOs, with a significant amount of cannabidiol (85.03%) identified in the fresh stembark oil of the Lifter cultivar (DLSO). Meanwhile, Dronabinol, also known as Tetrahydrocannabinol (THC), was present in the dried stembark oil in lesser amounts. The observed THC concentration (0.86–2.86%) exceeded the European Union (EU) regulation limit of 0.2% for cannabis plants used for industrial purposes [31]. However, this suggests that the Lifter cultivar is a high-CBD-yielding variety. Cannabinol, found in LSO and DCSO, is thought to be a breakdown product of THC during storage [38]. The oxygenated sesquiterpenes such as α-Bisabolol (2.01–2.73%), Bulnesol (0.35%), 5-epi-7-epi-α-Eudesmol (0.85%), and α-Eudesmol (0.35%) present in significant amounts in the Lifter stembark oils, have been reported to have antifungal, antiplatelet, anticholinesterase, antioxidant, antiemetic, anti-inflammatory, anticancer, and anti-tumor activities [25,39,40]. Therefore, considering the previous biological reports on the oil constituents of *C. sativa*, perhaps, the stembark Lifter cultivar of *C. sativa* grown in Komga, South Africa, may be a potential source of therapeutic agents for disease management.

### 2.3. Evaluation of In Vitro Antioxidant Activity of the Fresh and Dried C. sativa Stembark EOs of Two C. sativa Cultivars

**Figure 1:**
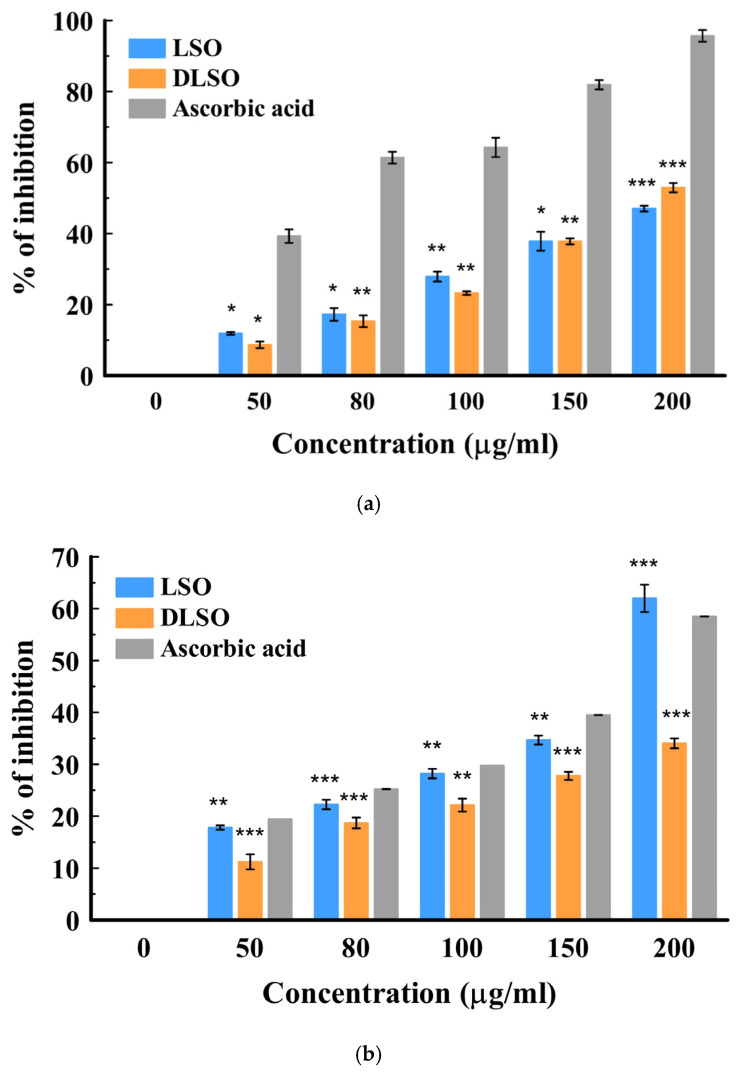
(**a**): In vitro antioxidant activity of fresh (LSO) and dried (DLSO) stembark oil by the H_2_O_2_ method. Data represented as mean ± SD (*n* = 3); * *p* ≤ 0.05; ** *p* ≤ 0.01; and *** *p* ≤ 0.001 when compared with the standard ascorbic acid. (**b**): In vitro antioxidant activity of fresh (LSO) and dried (DLSO) stembark oil using the DPPH method. Data are presented as mean ± SD (*n* = 3); ** *p* ≤ 0.01; and *** *p* ≤ 0.001 when compared with the standard ascorbic acid.

**Figure 2:**
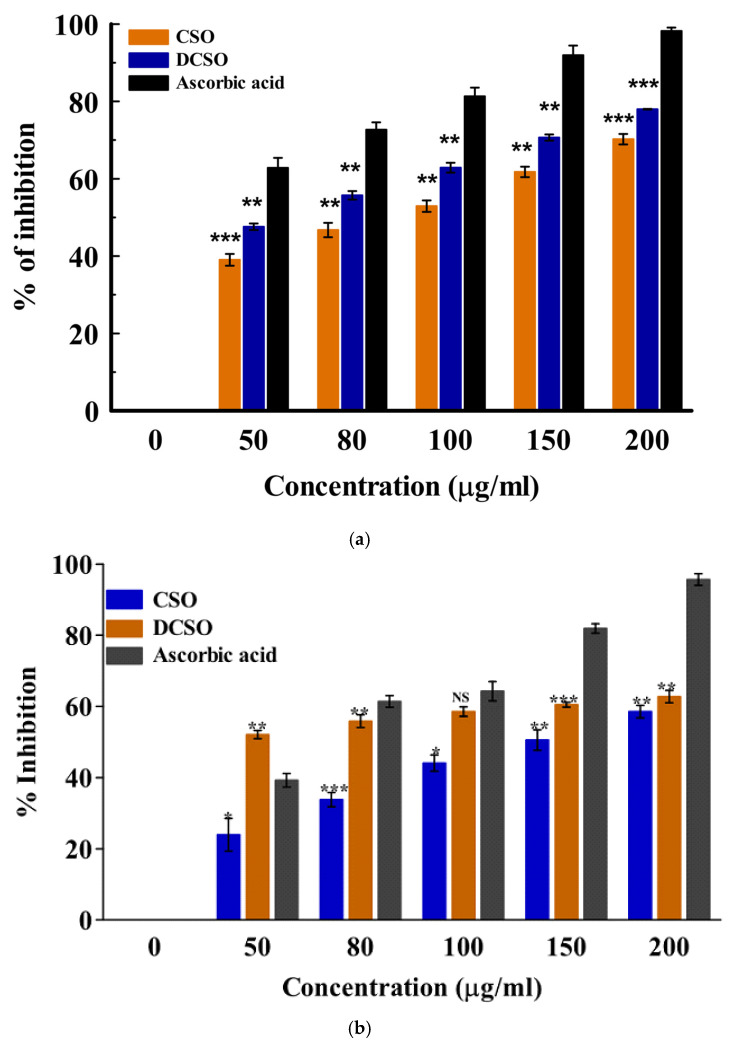
(**a**): In vitro antioxidant activity of fresh (CSO) and dried (DCSO) stembark oil determined by the DPPH method. Data represented as mean ± SD (*n* = 3); ** (*p* ≤ 0.01); *** (*p* ≤ 0.001). (**b**): In vitro antioxidant activity of fresh (CSO) and dried (DCSO) stembark oil of the Cherry-wine cultivar using the H_2_O_2_ method. Data represented as mean ± SD (*n* = 3); NS = no significant difference (*p* > 0.05); * (*p* ≤ 0.05); ** (*p* ≤ 0.01); *** (*p* ≤ 0.001).

The antioxidant activity of the essential oils was assessed using two spectrophotometric assays: DPPH and H_2_O_2_. These methods measure the ability of the natural antioxidant compound to react with the DPPH radical (colored probe) or quench the peroxyl radicals. There are two main mechanisms of action involved in antioxidant effects: hydrogen atom transfer and single electron transfer [41]. Hydrogen atom donation from the antioxidant agent (compound) deactivates the radical, while in the electron transfer process, the antioxidant agent transfers one electron to deactivate the free radical [41]. The result showed a concentration-dependent increase in the mean percentage inhibition from 50–200 µg/mL in both cultivars. The radical scavenging activities of the fresh (LSO) and dried (DLSO) *C. sativa* Lifter oils are shown in Figure 1a,b. In the H_2_O_2_ method, DLSO and LSO showed good antioxidant activity at 200 µg/mL. However, this activity was lower than the standard ascorbic acid. The radical scavenging activity of the essential oils from the fresh and dried stembark oils of the Cherrywine cultivar is shown in Figure 2a,b. At the highest concentration (200 µg/mL), DCSO showed significantly (*p* < 0.001) better antioxidant activity against the DPPH radical compared to CSO. Additionally, DCSO exhibited significantly (*p* < 0.001) higher mean percentage inhibition across all concentrations compared to CSO. Further antioxidant evaluation using the DPPH assay suggests that DCSO is a better oil. It demonstrated better inhibition of the DPPH radical across all concentrations. In the H_2_O_2_ assay (Figure 2b), DCSO demonstrated ≈ 60% inhibition at 100 µg/mL, which was comparable to the standard drug at the same concentration. It also proved to be a more antioxidant--active oil compared to CSO across all concentrations. Conversely, the lower antioxidant activity of CSO in the DPPH and H_2_O_2_ assays might be attributed to a lower chemical composition. The overview of the antioxidant activity of the two cultivars is presented in Table 3 in the form of the 50% inhibitory concentration (IC_50_) values. The results show that the oil from the dried Lifter cultivar (DLSO) exhibits the best antioxidant activity among the EOs based on its lowest IC_50_ values of 21.68 ± 1.71 and 26.20 ± 1.34 µg/mL against DPPH and H_2_O_2_, respectively.

A study of two varieties of *C. sativa* (Futura 75 and Felina) cultivated in Lithuania evaluated the antioxidant activity by H_2_O_2_ scavenging activity of the stem extract using a kinetic approach. A decrease in the hydrogen peroxide-induced reduction current indicated that the hydrogen peroxide was scavenged by the stem extract [42]. The study recorded α-Pinene (20.25%) as the major constituent, and monoterpenes (31.38%) dominated the stem oil. Oil samples from different varieties of *C. sativa* inflorescences cultivated in Valparaíso, Chile, have been reported to possess DPPH scavenging activity, with IC_50_ values of 12.18–18.38 mg/mL [43]. Quantified cannabinoids present in the oils include CBD (508– < 1.5 mg/g) and THC (677- 6.5 mg/g) with high total content of phenols of 102.07 ± 1.70 mg/g, total flavonoids (9.10 ± 0.06 mg/g) and total anthraquinone of 6.32 ± 0.03 mg/g. *C. sativa* extracts (aqueous and hexane extracts) of a hemp inflorescence (Futura 75), cultivated in Fiuminata, central Italy have also been shown to exhibit IC_50_ values of 60 and 97 µg/mL for DPPH scavenging capacity of aqueous and hexane extract, respectively, and 9 and 127 µg/mL for superoxide radical scavenger activity using ascorbic acid as the reference drug [44]. A low amount of CBD (0.85 mg/g) was detected in the aqueous extract with a high amount of luteolin-7-O-glucoride and apigenin glucuronide (31.1 and 15.4 mg/g, respectively). Hexane extract had high CBD (160.5 mg/g) and CBDA (14.4 mg/g) contents.

A lower IC_50_ value of <50 µg/mL indicates strong antioxidant activity [45,46,47,48] and that the drug or plant extract may be effective, resulting in lower systemic toxicity when administered to a patient at low concentration [49]. Consequently, it was observed from the DPPH and H_2_O_2_ assay methods used in this study that the Lifter *C. sativa* cultivar stembark oils show strong antioxidant activities (21.68, 26.20, 29.26, and 38.60 µg/mL) when compared to the Cherrywine stembark oils. However, their higher IC_50_ values compared to the standard drug suggest that the oils possess lower potency compared to the standard drug. Thus, the displayed antioxidant activities of the oils may be attributed to both electron transfer and hydrogen atom transfer from the terpenoid, phytocannabinoid, and fatty acid components of the EOs to the oxidants, leading to observable color change in the DPPH and H_2_O_2_ experiments. The notable antioxidant activity of the dried Lifter stembark oil may be associated with its higher number (30) of constituents (broader chemical profile) compared to the fresh oil, which showed 11 constituents. A significant correlation exists between the presence of terpenoids, fatty acids, and phytocannabinoid concentration of *C. sativa* and antioxidant activity [44,50]. Thus, an explanation for the higher antioxidant activity seen in the Lifter cultivar when compared to the Cherrywine cultivar (for both methods of assays) could be that the amount of cannabidiol (85.03%) present in the stembark oil of the Lifter cultivar contributed significantly to the scavenging activity of the oil. Perhaps, the LSO with 85% Cannabidiol content may have the potency to scavenge the DPPH and H_2_O_2_ free radicals [51]. However, a more in-depth compositional or bioactivity correlation analysis would be required to establish a stronger basis for these observations. The antioxidant profile of the cultivars studied could also be influenced by harvest, cultivar conditions, and the synergistic effect between the oil constituents. Additionally, our study on the two *C. sativa* cultivars further underscores the importance of the geographical location of cultivation on the EO composition and antioxidant activities. This is important because of the wide range of varietals existing in *C. sativa*, representing a large number of cultivars across the globe. [43,44]. Although genetic analysis was beyond the scope of this study, it is important to note that minor genetic or epigenetic variability, particularly in a hybrid cultivar (Cherrywine), may influence phytochemical composition and antioxidant potential. Significant variation among individual plants, likely influenced by epigenetic differences rather than DNA sequence variation, was reported in the case of *Arabidopsis thaliana* [52]. Other studies have also provided evidence for the interplay of genetic and epigenetic variation in shaping molecular and non-molecular phenotypes [53,54,55,56]. Thus, genetic or epigenetic profiling would further clarify any underlying genetic factor(s) capable of modulating chemical composition and antioxidant activity capacity of the cultivars.

### 2.4. In Silico Molecular Docking of Stembark Oil Constituents of Two C. sativa Cultivars

**Figure 3:**
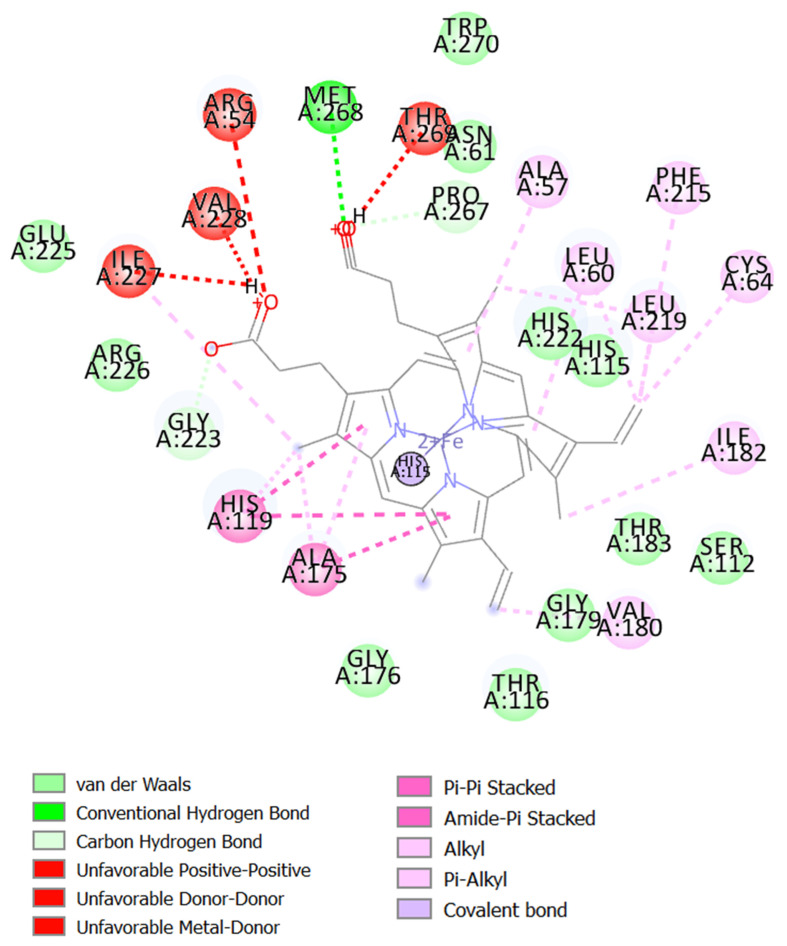
The inhibitor (native ligand) is bound to the binding pocket of NOX2 and amino acid interactions.

**Figure 4:**
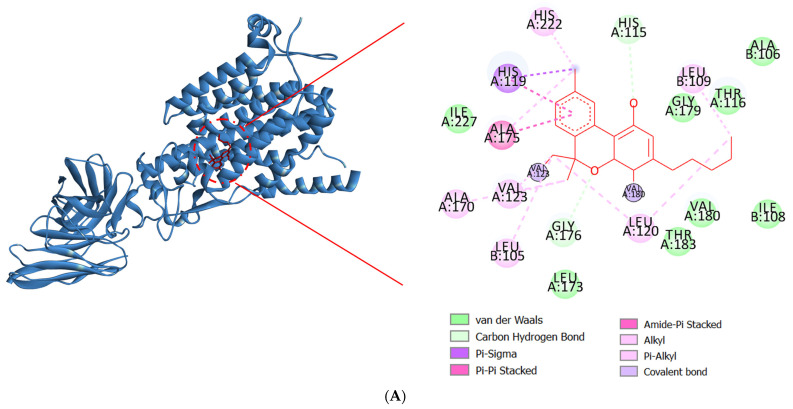

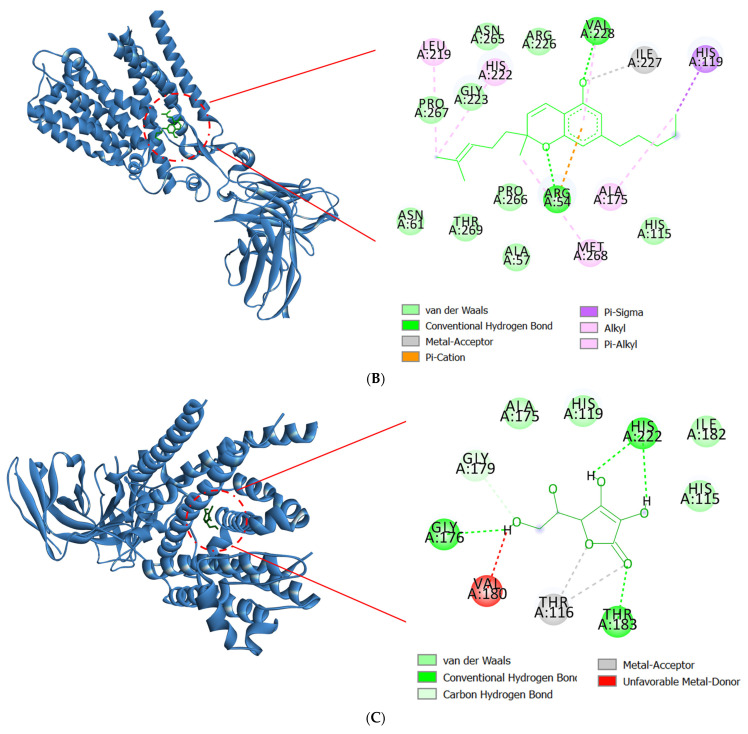
Molecular docking interactions of NOX2 with (**A**) Cannabinol, (**B**) Cannabichromene, and (**C**) L-ascorbic acid. The blue ribbon represents the NOX2 protein.

Drug discovery frequently uses molecular docking studies to forecast how small compounds (ligands) might attach to the NOX2 protein complex. A crucial enzyme in the production of reactive oxygen species (ROS), which are implicated in a number of biological processes and diseases, is NOX2, often referred to as NADPH oxidase 2. When the enzyme NOX moves an electron from one oxygen atom to another, superoxide is created. CAT uses reduced GSH to further catalyze hydrogen peroxide conversion, which first converts from superoxide to water [57]. This process is implicated in various neurodegenerative illnesses, such as Alzheimer’s and Parkinson’s, stroke, traumatic brain injury, and acute and chronic pulmonary inflammatory diseases. Docking simulates the binding of various substances to the protein’s active site, which aids in the identification of possible NOX2 inhibitors [58]. The major constituents identified in fresh and dried stembarks of the Lifter and Cherrywine *C. sativa* cultivars were molecularly docked against the NOX2 protein. In this investigation, we examined how the suggested inhibitor (native ligand) protoporphyrin IX containing Fe bound to the NOX2 protein and contrasted it with the phytochemicals’ binding patterns that were of interest. With a binding energy value of −11.0 kcal/mol, the inhibitor (native ligand) demonstrated a substantial binding potential and implicated the residues at NOX2’s active binding pocket (Figure 3). Table 4 displays the binding energy scores of the constituents from the Lifter cultivar against the NOX2 protein. Among the thirty-two phyto-compounds, Cannabinol showed the best binding energy score of −9.7 kcal/mol with hydrophobic interactions (Ala106, Ile108, Thr116, Leu173, Gly179, Val180, Thr183, Ile227), hydrogen bonds (His115, Gly176), Pi-Sigma (Ala175), and Pi-Alkyl interactions including Leu105, Leu109, Leu120, Val123, Ala170, His222 (Figure 4A). Other promising constituents were Cannabidiol and Linalool with binding energy scores of −8.5 and −6.5 kcal/mol, respectively. On the other hand, Table 5 displays the binding energy scores of the forty-two phytocompounds from the Cherrywine cultivar against the NOX2 protein. A binding energy score of −8.8 kcal/mol was seen in the interaction of Cannabichromene (only present in dried CSO) with hydrophobic interactions (Ala57, Asn61, His115, Gly223, Arg226, Asn265, Pro266, Pro267, Thr269), hydrogen bonds (Arg54 and Val228), Pi-Alkyl interactions (Ala175, Leu219, His222, Met268), and Pi-Sigma interaction (His119) (Figure 4B). The standard L-ascorbic acid showed a binding energy score of −5.7 kcal/mol with hydrophobic interactions (His115, His119, Ala175, Ile182) and hydrogen bonds (Gly176, Gly179, Thr183, His222) (Figure 4C). The active site residues His115, Thr116, His119, Ala175, Gly176, Gly179, Val180, His222, and Ile227 of cannabinol-NOX2, residues and Asn61, Ala57, His115, Gly223, Arg226, Pro266, Pro267, Thr269, Ala175, Leu219, His222, Met268 of Cannabichromene-NOX2, and residues His115, Thr116, His119, Ala175, Gly176, Gly179, Val180, Ile182, Thr183, and His222 of L-ascorbic acid-NOX2 were involved in the interactions, which is essential for binding the putative inhibitor. As a result, it seems sensible that Cannabinol from Lifter and Cannabichromene from Cherrywine should be able to bind and inhibit NOX2 quite effectively. Thus, molecular docking is a useful technique for comprehending how small compounds interact with the NOX2 protein, which may help find novel NOX2 inhibitors to treat a range of illnesses.

## 3. Materials and Methods

### 3.1. Reagents and Experimental Procedure

HPLC-grade solvent (hexane, 2.5 L), DPPH (1,1-diphenyl-2-picrylhydrazyl), hydrogen peroxide, glasswares, and vials were sourced from Sigma-Aldrich (Pty) Ltd. (Johannesburg, South Africa), through a licensed local supplier, Shalom Laboratories (Musgrave Road, Durban, South Africa). Distilled water was prepared and used for hydro-distillation. A Clevenger-type apparatus was employed. Absorbance values of the DPPH and H_2_O_2_ assays were obtained on a 680- Bio-Rad microplate reader (serial number 14966, Irvine, CA, USA).

### 3.2. Ethical Consideration

A Permit for research on *Cannabis sativa,* including cannabis collection, was obtained from the South African Health Products Regulatory Authority (SAHPRA) under permit No. PIA-HP-EC-2022-0023. Furthermore, approval for the execution of the study was obtained through the Walter Sisulu University research ethics committee with approval reference number WSU/FNS-GREC-2021/12/01/G9. Fresh whole cannabis plants of the two cultivars studied were also deposited at Walter Sisulu University’s herbarium for authentication, with voucher numbers AEO-001 and AEO-002 assigned to the Lifter and Cherrywine cultivars, respectively.

### 3.3. Sample Collection and Extraction Procedure

**Figure 5:**
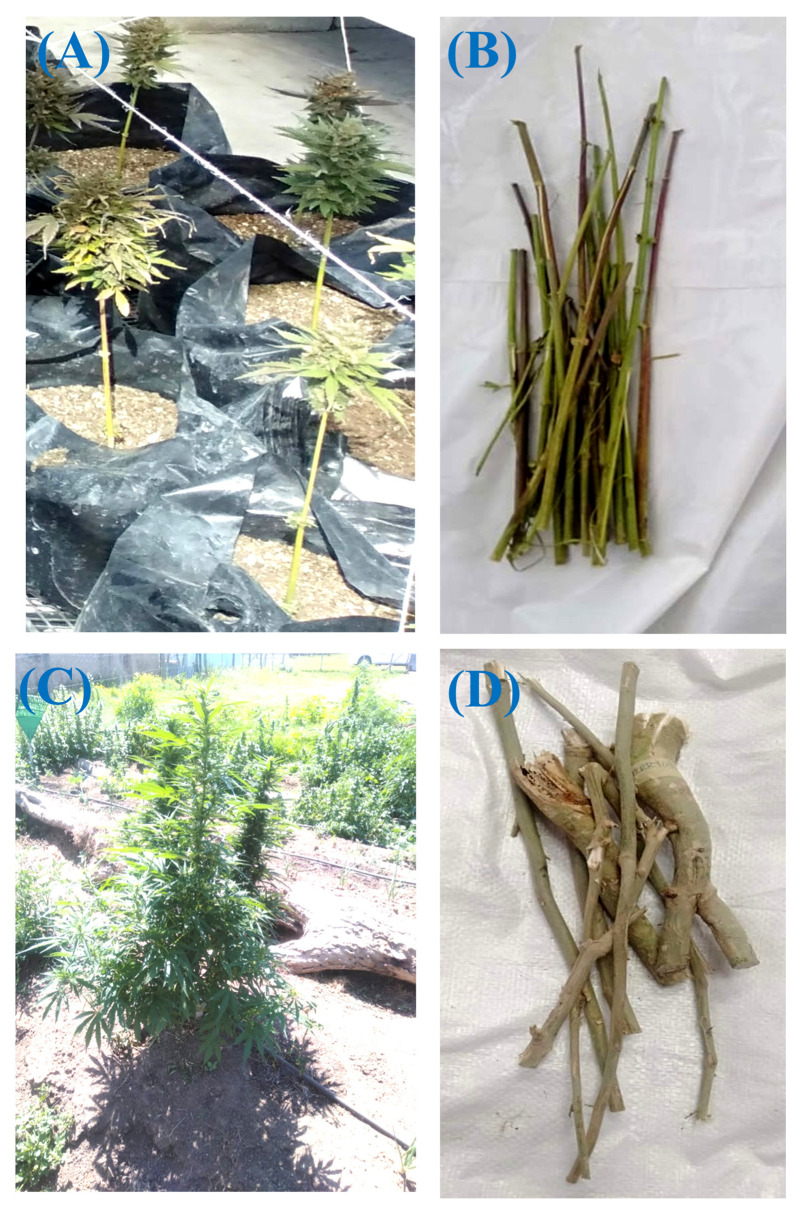
Lifter whole plant (**A**); fresh Lifter stems (**B**); Cherrywine whole plant (**C**); fresh Cherrywine stems (**D**).

The cultivars were obtained from the Eastern Cape Hemp Producers Association (ECHPA) in South Africa. The seeds of the Lifter cultivar with serial number Lot#:2020—-A6, were sourced from Jack Hempicine LLC, 3395 S Pacific Hwy. W, Independence, OR, USA (OR 97351) and were cultivated using the greenhouse approach, while Cherrywine was sourced from Bodhi Urban of Longmont, Longmont, CO, USA (CO 80501) and was cultivated outdoors. Notably, Cherrywine is a hybrid of 50% indica and 50% sativa, while the Lifter cultivar is 100% sativa. The morphological difference(s) of both cultivars are shown in Figure 5.

Dried (shed-dried in the dark at 25 °C for 14 days) and fresh stembarks of the two *C. sativa* cultivars were obtained in sealed FDA-approved polyethene bags from Komga in the Eastern Cape province of South Africa (GPS coordinates: 32.577 °S 27.888 °E). Extraction of EOs from fresh and dried stem bark samples was carried out following ASTM (D8282-19) guidelines [59] and according to the methods of Naz et al. [29] and Oyedeji et al. [60] with slight alterations. A hydro-distillation method using a Clevenger-type apparatus was used to extract essential oils from the stembarks of the cultivars. The fresh stembark samples were extracted immediately after they were brought from the farm, while the dried samples were extracted subsequently. The weight of the fresh and dried stembarks (LSO, DLSO, CSO, and DCSO) ranged from 23 g to 136 g. A sample-to-solvent ratio of 1:15 was used to ensure the level of the solvent (distilled water) was above the plant material placed in a round-bottom flask. The round-bottom flask was placed on the heating mantle, and the temperature was set to 100 °C for 1 hr and then decreased to 80 °C for 3 hrs., for a total extraction time of 4 hrs. The EO was collected over hexane and water. The EO accumulated in the hexane portion was collected and stored in glass vials at 0–4 °C until analysis.

### 3.4. Physicochemical Analysis of the EOs

The yields of the EOs were determined gravimetrically (*w*/*w*) as percentage (%) yield of fresh (LSO, CSO) and dried (DLSO, DCSO) stembark of the *C. sativa* cultivars using Equation (1):

(1) $$\mathrm{Essential}\;\mathrm{oil}\;\mathrm{yield}\;(\%)\;=\;\frac{W1}{W2}\times 100$$

where W1 = net weight of oils (grams) and W2 = total weight of fresh/dried plant (grams). The oil yield is reported as (% *w*/*w*). Gas chromatography coupled with mass spectrometry (GC-MS) was used to determine the chemical composition (terpene profiles) of the essential oils extracted.

### 3.5. GC-MS Sample Preparation and Analysis

Chromatographic separation was performed on a gas chromatograph (6890 N, Agilent Technologies Network) coupled to an Agilent Technologies Inc., Palo Alto, CA, USA, inert XL EI/CI Mass Selective Detector (MSD) (5975B, Agilent Technologies Inc., Palo Alto, CA, USA). The GC-MS system was coupled to a CTC Analytics PAL autosampler. Separation of the essential oils was performed on a 5% phenyl dimethylpolysiloxane fused-silica non-polar ZB-5MS (30 m, 0.25 mm ID, 0.25 μm film thickness) capillary column Model Number: Zebron 7HG-G010-41. Helium was used as the carrier gas at a flow rate of 1 mL/min. A quantity of 50 μL of the sample was diluted with 950 μL of acetone before injection into the GC-MS. A quantity of 1 μL of EOs of *C. sativa* cultivars was diluted in 4 μL *n-hexane* and the mixture was injected into the GC operated at a 50:1 split ratio. The injector temperature was maintained at 240 °C. The oven temperature was programmed as follows: 50 °C for 6 min and ramped at a rate of 5 °C/min until 320 °C and held for 2 min. The MSD was operated in a full scan mode, and the source and quad temperatures were maintained at 230 °C and 150 °C, respectively. The transfer line temperature was maintained at 250 °C. The mass spectrometer was operated under electron impact (EI) mode at an ionization energy of 70 eV, scanning from 35 to 650 *m*/*z*.

#### Identification and Quantification of the EOs

The chemical compounds were quantified based on the relative response in the MS chromatograms and identified through their linear retention indices (LRI) relative to a series of n-alkanes (C_9_–C_36_) on the same capillary column. LRI values obtained were compared with values reported in the NIST web book, ChemSpider, PubChem, and other literature [31,60,61,62,63].

### 3.6. Determination of Antioxidant Activity

#### 3.6.1. DPPH Radical Scavenging Assay

DPPH free radical scavenging activity of the EOs was determined following a previously reported method [64] with slight modification. A total volume of 40 mL of 0.1 mM DPPH free radical in methanol was added to 1 mL (1000 μL) serially diluted oil sample at 200, 150, 100, 80, and 50 μg/mL concentrations in triplicate. A quantity of 200 μL of test samples and control solution were transferred into a 96-well plate and incubated in the dark at room temperature for 30 min. The absorbance was measured at 515 nm on a microplate reader. Radical scavenging activity was calculated according to Equation (2):

(2) $$\%\;\mathrm{DPPH}\;\mathrm{inhibition}\;=\;\frac{ABScontrol-ABSsample}{ABScontrol}\times 100$$

where ABSsample = absorbance of the test sample, while ABScontrol = absorbance of the negative control (methanol). The data obtained was plotted against the concentration of the oil samples, and the fitting of the data to the dose-response equation provided the IC_50_ values, which shows the concentration that caused a 50% inhibition of the DPPH radical.

#### 3.6.2. Hydrogen Peroxide (H_2_O_2_) Scavenging Assay

The ability of the stembark oil samples to inhibit hydrogen peroxide free radicals was determined using a standard calorimetric method [65]. The EOs of the two *C. sativa* cultivars (1100 μL each) were serially diluted in methanol to 200, 150, 100, 80, and 50 μg/mL concentrations in triplicate. Briefly, 22 μL (2.16 mM) of H_2_O_2_ was mixed with 100 mL of H_2_O. Then, 600 μL of prepared hydrogen peroxide solution was added to the samples and the control. The reaction was incubated at room temperature (25 °C) for 10 min, and absorbance was determined at 595 nm. The percentage inhibition of the hydrogen peroxide radical was calculated, using Equation (3):

(3) $$\%\;\mathrm{inhibition}\;\mathrm{of}\;\mathrm{radical}\;\mathrm{of}\;H_{2}O_{2}\;=\;\frac{ABScontrol-ABSsample}{ABScontrol}\;\times \;100\;$$

where ABSsample = absorbance of test samples/ascorbic acid, while ABScontrol = absorbance of negative control (H_2_O).

#### 3.6.3. Statistical Analysis

The antioxidant assay result was analyzed using KyPlot software version 6.0 (Kyenslab, Tokyo, Japan). Differences between the standard and EO samples were compared for significance using a parametric test (*t*-test for paired comparison) for paired samples. All measurements are expressed as mean ± standard deviation (*n* = 3). Linear square regression was selected to calculate IC_50_ values. Differences considered significant (*p* < 0.05) were labeled with an asterisk (*) within the bar graphs.

### 3.7. In Silico Molecular Docking

#### 3.7.1. Preparation and Refinement of the Protein and Ligand Structures

Molecular docking analysis was conducted using the main chemicals found in fresh and dried stembarks of Lifter and Cherrywine cultivars of *C. sativa* against oxidative stress. The Protein Data Bank (http://www.rcsb.org (accessed on 25 June 2025)) provided the PDB structures of NOX2, which have a PDB ID of 7U8G [66]. After removing the water atoms from the protein structures, AutoDockTools was used to add polar hydrogen atoms and Kollman charges in preparation for docking. The NCBI PubChem (https://pubchem.ncbi.nlm.nih.gov/ (accessed on 25 June 2025)) was used to download the phyto-compounds. The sdf structures that were downloaded were then converted into pdb structures using the Open Babel Server [67]. The ligand structures were optimized for energy using the PRODRG server [68], and then their energy was minimized using the Gromos 96 force field.

#### 3.7.2. Determination of the Active Site and Molecular Docking

The literature [66] was used to predict the active site of the protein (NOX2). The CASTp 3.0 (Computed Atlas of Surface Topography of Proteins) online server [69] was used to validate the predictions. The top result from the top three possible ligand-binding sites was selected for docking after the processed protein without a native inhibitor was submitted to the CASTp 3.0 server. Then, the amino acids at the active sites of the native inhibitor-NOX2 co-crystallized complex were compared with the amino acid residues predicted by CASTp 3.0. To confirm the active site, the co-crystallized complex was manually opened in the Discovery Studio visualization tool [70]. As a result, the interacting residues could be identified and were discovered to be fairly close to those that the CASTp 3.0 server had predicted. For each atom of the native inhibitor ligand, grid maps (X, Y, and Z confirmations; Box center and Box dimension) were created using the Autodock and Autogrid tools connected with Autodock4. Molecular docking was performed using Autodock4 to obtain the X, Y, and Z coordinates (Box center and Box dimension) as a possible target location. A stiff protein receptor and a flexible ligand docking methodology were used to molecularly dock the ligands of interest at the active binding sites of the relevant protein using a grid-based molecular docking technology [70,71]. The NOX2 protein’s active site residues were represented in a grid box with the following values: size_x = 21.0, size_y = 34.0, size_z = 35.0, center_x = 144.171, center_y = 141.09, and center_z = 151.26. The molecular docking process was completed by Autodock Vina, and the docked complexes were then visualized with the Discovery Studio visualization tool [70].

### 3.8. Study Flow Chart

**Figure 6:**
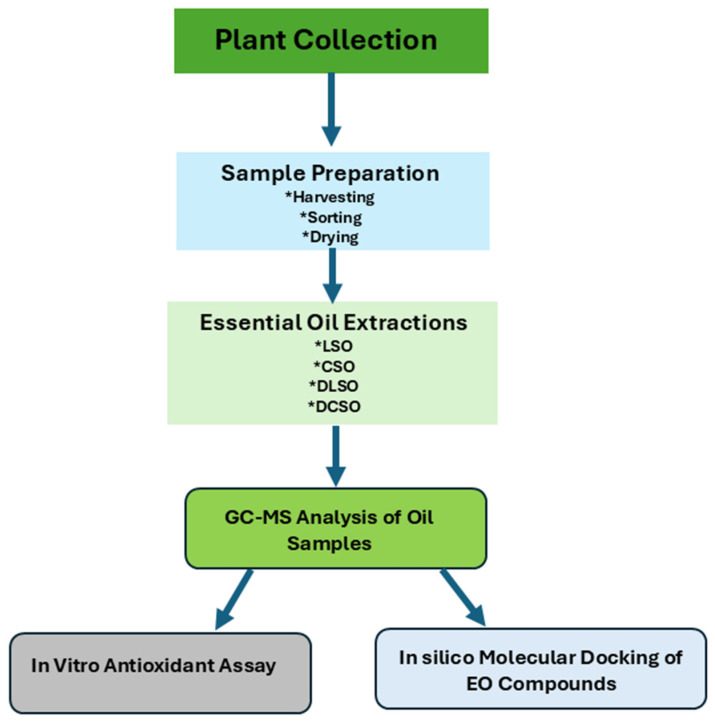
Schematic flowchart.

The schematic flow chart in Figure 6 outlines the study.

## 4. Conclusions

The stembarks of the two *C. sativa* cultivars from Komga in South Africa, often regarded as waste material, were investigated for their essential oils and antioxidant capacity. The dried stembark of the Cherrywine cultivar afforded a higher oil yield with more chemical constituents (55) than the oil from the fresh stembark (CSO) and the fresh (LSO) and dried (DLSO) stembark oils of the Lifter cultivar. In addition, the outdoor-cultivated Cherrywine afforded unique essential oil constituents including Zingiberenol, α-Calacorene, Caryophylla-4(12),8(13)-Dien-5-α-ol, α-Cubebene, α-Curcumene, and Bicyclo-[7.2.0]undecan-3-ol, 11,11-dimethyl-4,8-bis(methylene). Sesquiterpenes dominated the essential oils of the two cultivars. Neutral cannabinoids were also detected in the essential oils, with a significant amount of Cannabidiol found in the fresh Lifter stem oil. The EO from the dried Lifter stembark oil (DLSO) showed better antioxidant activity than the other stembark oils based on their considerable DPPH and H_2_O_2_ radical scavenging effects. The displayed activity of the DLSO may be attributed to the exclusive presence of monoterpene, oxygenated, and hydrocarbon sesquiterpenes in the oil. The putative compounds include Terpineol, (+/−)-β-cadinene, Selina-3,7(11)-diene, Isoaromadendrene epoxide, 5-epi-7-epi-α-Eudesmol, Bulnesol, Juniper camphor, Phytol (trans), Pentacosane, Hexacosane, Heptacosane, and Squalene. Further in silico analysis revealed Cannabinol, Cannabidiol, and Linalool as promising constituents based on their high binding energy scores with NOX2, a protein that has been implicated in oxidative damage. This study has, therefore, highlighted the considerable antioxidant activity of the *C. sativa* stembark oils and their potential in the management of oxidative stress-related conditions. A limitation of this study is the lack of genetic analysis of the cannabis cultivars using genomic tools to clarify genetic variability and its potential influence on the performance of the cultivars. Future directions may also include evaluating the toxicity, including MTT cytotoxicity, total ROS analysis, and in vivo activity of the oils against oxidative damage to DNA, lipids, and proteins.

## Figures and Tables

**Table 1:** Percentage yield and physicochemical analysis of stembark EOs of two *C. sativa* cultivars.

Sample Codes	Total Weight of Fresh Plant (g)	Net Weight of Oils (g)	% Yield (*w*/*w*)	Color	Odor/Scent
LSO	22.73	0.12	0.53	Transparent	Mildly skunky
CSO	136.40	0.25	0.18	Transparent	Mildly sweet
DLSO	30.00	0.20	0.67	Pale yellow	Mildly sweet
DCSO	50.00	0.79	1.58	Transparent	Mildly sweet

Fresh (LSO; CSO) and dried (DLSO;DCSO) stembark oils of two *Cannabis sativa* cultivars.

**Table 2:** Chemical composition of EOs from fresh and dried stembarks of two cultivars of *C. sativa*.

RT (Min)	Chemical Components Identified ^a^	CAS	RI Cal. ^b^	RI Lit.	Percent Composition of Plant Parts (%)			
					LSO ^c^	CSO ^c^	DLSO ^d^	DCSO ^d^
8.815	α-Pinene	000080-56-8	936	936	_	_	_	0.16
11.172	β-Myrcene	000123-35-3	998	997	_	0.95	_	0.31
12.540	D-Limonene	005989-27-5	1033	1030	_	0.35	_	0.25
13.299	β-cis-Ocimene	003338-55-4	1053	1053	_	0.33	_	_
15.122	Linalool	000078-70-6	1101	1103	_	_	0.24	0.30
15.503	Fenchol	001632-73-1	1110	1123	_	_	_	0.13
17.994	Terpineol	1000411-59-6	1176	***	_	_	0.13	_
18.304	Dodecane	000112-40-3	1205	1200	_	_	_	0.11
21.207	Tridecane	000629-50-5	1305	1300	_	_	_	0.11
23.308	α-Cubebene	017699-14-8	1377	1349	_	_	_	0.11
24.125	Isocaryophyllene	000118-65-0	1410	1406	_	0.73	_	0.67
24.323	Cis-α-Bergamotene	018252-46-5	1418	1415	_	0.49	_	_
24.552	Caryophyllene	000087-44-5	1428	1423	_	16.61	0.64	7.90
24.717	(E)-β-Farnesene	028973-97-9	1435	1445	_	_	_	0.13
24.868	Trans-α-Bergamotene	013474-59-4	1441	1437	_	2.50	_	1.63
24.968	α-Guaiene	003691-12-1	1445	1439	_	2.69	_	1.37
25.378	Humulene	006753-98-6	1462	1457	_	8.15	0.37	4.47
25.521	9-epi-trans-Caryophyllene	068832-35-9	1468	1467	_	_	_	0.63
25.900	γ-Muurolene	030021-74-0	1483	1480	_	_	_	0.64
26.020	α-Curcumene	000644-30-4	1488	1480	_	0.35	_	0.14
26.091	Naphthalene, 1,2,3,4,4a,5,6,7-octahydro-4a,8-dimethyl-2-(1-methylethenyl)-;	103827-22-1	1491	1492	_	0.63	_	_
26.143	α-Selinene	000473-13-2	1493	1493	_	2.26	_	1.69
26.166	(+)-β-Selinene	017066-67-0	1494	1492	_	_	_	1.46
26.486	α-Amorphene	020085-19-2	1507	1485	_	_	_	0.26
26.677	δ-Guaiene	003691-11-0	1515	1509	_	5.65	_	2.82
26.743	β-Curcumenene	028976-67-2	1518	1518	_	_	_	0.19
26.824	γ-Cadinene	039029-41-9	1521	1513	_	_	_	0.7
27.040	(+)-δ-Cadinene	000483-76-1	1530	1524	_	_	_	0.87
27.337	γ-Selinene	000515-17-3	1542	1479	_	_	0.15	0.48
27.394	(+/−)-β-cadinene	005951-61-1	1544	***	_	_	0.18	_
27.505	Selina-3,7(11)-diene	006813-21-4	1549	1546	_	_	0.38	_
27.511	α-Calacorene	021391-99-1	1549	1542	_	_	_	0.23
27.759	Sesquisabinene hydrate, cis	058319-05-4	1559	1540	_	_	_	0.29
28.011	Nerolidol	000142-50-7	1570	1570	_	4.99	0.32	2.74
28.582	Caryophyllene oxide	001139-30-6	1593	1583	1.27	19.58	1.74	9.41
28.888	Guaiol	000489-86-1	1605	1600	0.64	0.37	0.66	_
28.969	Ledol	000577-27-5	1609	1608	_	_	0.18	0.21
29.167	Humulene-1,2-epoxide	019888-34-7	1617	1603	0.45	5.78	0.81	3.32
29.409	7-epi-γ-Eudesmol	117066-77-0	1627	1660	0.74	0.53	1.00	_
29.506	Isoaromadendrene epoxide	1000159-36-6	1632	1590	_	_	0.35	_
29.539	Zingiberenol	058334-55-7	1632	1611	_	_	_	0.75
29.608	Trans -α-Bisabolene epoxide	1000131-71-1	1635	1586	_	1.52	_	_
29.704	Caryophylla-4(12),8(13)-dien-5-α-ol	019431-79-9	1639	1637	_	_	_	1.00
29.727	Bicyclo [7.2.0]undecan-3-ol,11,11-dimethyl-4,8-bis(methylene)-	079580-01-1	1640	1645	_	_	_	1.50
29.774	Caryophylla-4(12),8(13)-dien-5-β-ol	019431-80-2	1642	1644	_	2.33	_	_
30.034	β-Eudesmol	000473-15-4	1652	1652	0.34	0.50	0.50	_
30.090	5-epi-7-epi-α-Eudesmol	1000411-50-1	1655	1616			0.85	_
30.177	α-Eudesmol	000473-16-5	1658	1643	0.39	_	_	_
30.405	Bulnesol	022451-73-6	1668	1666	_	_	0.35	_
30.772	α-Bisabolol	000515-69-5	1683	1685	2.01	_	2.73	_
31.046	Juniper camphor	000473-04-1	1694	1700	_	_	0.27	_
32.401	Myristic acid	000544-63-8	1731	1763	_	_	_	0.26
32.471	Benzyl Benzoate	000120-51-4	1734	1763	_	_	_	0.53
34.081	Hexahydrofarnesyl acetone	000502-69-2	1848	1844	_	_	0.27	0.40
34.393	Pentadecanoic acid	001002-84-2	1834	1867	_	_	0.16	0.28
34.608	Benzyl salicylate	000118-58-1	1816	1860	_	_	_	0.34
34.798	n-Cetyl alcohol	036653-82-4	1865	1880	_	_	_	0.22
36.053	Palmitoleic acid	000373-49-9	1919	1941	_		_	0.29
36.728	Palmitic acid	000057-10-3	1948	1962	1.45	_	3.99	4.45
37.612	Isopropyl palmitate	000142-91-6	2026	2025	_	_	_	0.37
39.243	Phytol (trans)	000150-86-7	2115	2116	_	_	0.30	_
39.655	Linoleic acid	000060-33-3	2116	2113	_	_	0.22	0.60
39.684	cis-13-Octadecenoic acid	013126-39-1	2117	2178	_	_	_	0.62
39.749	cis-Vaccenic acid	000506-17-2	2120	2117	_	_	_	0.42
40.134	Stearic acid	000057-11-4	2139	2177	_	_	_	0.16
44.143	Tetracosane	000646-31-1	2406	2400	_	_	2.91	1.75
44.679	Cannabichromene	020675-51-8	2418	2440	_	_	_	0.17
44.825	Cannabidiol	013956-29-1	2426	2431	85.03	_	27.16	0.25
45.163	Cannabicoumaronone	070474-97-4	2444	2490	_	_	_	0.67
45.735	Pentacosane	000629-99-2	2508	2500	_	_	5.16	_
46.095	Dronabinol	001972-08-3	2496	2470	2.86	_	0.86	2.67
47.086	Cannabinol	000521-35-7	2550	2538	0.54	_	_	1.64
47.264	Hexacosane	000630-01-3	2609	2600	_	_	6.39	_
48.739	Heptacosane	000593-49-7	2710	2700	_	_	7.50	_
50.139	Octacosane	000630-02-4	2810	2800	_	_	6.55	3.37
50.500	Squalene	000111-02-4	2831	2833	_	_	0.31	_
52.250	2-Methylnonacosane	001560-75-4	2966	2958	_	_	_	0.19
	Total identified compounds				95.72	77.29	73.63	66.63
	Total Monoterpenes (%)				_	1.63	0.37	1.15
	Total Sesquiterpenes (%)				5.84	75.66	11.48	45.61
	Others (%)				89.88	_	61.78	19.87

^a^ Compounds listed in order of their retention time (RT). ^b^ RI (retention index) measured relative to n-alkanes (C_9_–C_36_) using a ZB-5MS capillary column. ^c^ Essential oil of fresh plant parts of Cultivar I (Lifter). ^d^ Essential oil of fresh plant parts of Cultivar II (Cherrywine). *** No literature for RI.

**Table 3:** In vitro antioxidant activities (IC_50_) of stembark oils of two *C. sativa* cultivars.

Test Samples	IC_50_ ± SD (µg/mL)	
	DPPH	H_2_O_2_
LSO	38.60 ± 0.48 ***	29.26 ± 1.80 *
DLSO	21.68 ± 1.71 *	26.20 ± 1.34 *
CSO	90.15 ± 8.32 ***	68.66 ± 9.90 ***
DCSO	64.18 ± 10.20 ***	55.06 ± 10.23 ***
Ascorbic acid	17.23 ± 0.13	21.43 ± 0.27

Data are expressed as mean ± standard deviation (*n* = 3). * (*p* ≤ 0.05); *** (*p* ≤ 0.001) of fresh Lifter (LSO), dried Lifter (DLSO), fresh Cherrywine (CSO), and dried Cherrywine (DCSO) stembark oils when compared to the standard L-ascorbic acid.

**Table 4:** Binding energy scores of the essential oil compounds from the dried and fresh stembark of *C. sativa* Lifter cultivar with NOX2 protein.

Sl. No.	Compound Name	Binding Affinity (Kcal/mol)
1.	Linalool	−6.5
2.	Terpineol	−5.5
3.	Caryophyllene	−5.8
4.	Humulene	−6.1
5.	γ-Selinene	−4.9
6.	(+/−)-β-cadinene	−4.5
7.	Selina-3,7(11)-diene	−5.7
8.	Nerolidol	−4.6
9.	Caryophyllene oxide	−4.2
10.	Guaiol	−4.2
11.	Humulene-1,2-epoxide	−4.3
12.	7-epi-γ-Eudesmol	−5.1
13.	β-Eudesmol	−5.5
14.	5-epi-7-epi-α-Eudesmol	−5.3
15.	α-Eudesmol	−6.2
16.	Bulnesol	−5.1
17.	α-Bisabolol	−5.2
18.	Juniper camphor	−4.3
19.	Hexahydrofarnesyl acetone	−4.7
20.	Pentadecanoic acid	−4.1
21.	Palmitic acid	−4.3
22.	Phytol (trans)	−4.0
23.	Linoleic acid	−3.9
24.	Tetracosane	−3.8
25.	Cannabidiol	−8.5
26.	Pentacosane	−4.1
27.	Dronabinol	−4.8
28.	Cannabinol	−9.7
29.	Hexacosane	−4.6
30.	Heptacosane	−5.7
31.	Octacosane	−5.1
32.	Squalene	−4.8

**Table 5:** Binding energy scores of the essential oil compounds from the dried and fresh stembark of *C. sativa* Cherrywine cultivar with NOX2 protein.

Sl. No.	Compound Name	Binding Affinity (Kcal/mol)
1.	α-Pinene	−7.0
2.	β-Myrcene	−6.4
3.	D-Limonene	−7.1
4.	β-cis-Ocimene	−6.5
5.	Fenchol	−7.0
6.	Dodecane	−6.0
7.	Tridecane	−6.3
8.	α-Cubebene	−8.3
9.	Isocaryophyllene	−8.2
10.	Cis-α-Bergamotene	−8.0
11.	(E)-β-Farnesene	−7.5
12.	Trans-α-Bergamotene	−8.2
13.	α-Guaiene	−8.0
14.	9-epi-trans-Caryophyllene	−7.7
15.	γ-Muurolene	−7.8
16.	α-Curcumene	−8.0
17.	Naphthalene, 1,2,3,4,4a,5,6,7-octahydro-4a,8-dimethyl-2-(1-methylethenyl)-	−7.8
18.	α-Selinene	−7.9
19.	(+)-β-Selinene	−6.5
20.	α-Amorphene	−7.6
21.	δ-Guaiene	−8.4
22.	β-Curcumenene	−8.0
23.	γ-Cadinene	−7.9
24.	(+)-δ-Cadinene	−7.1
25.	α-Calacorene	−7.8
26.	Sesquisabinene hydrate, cis	−7.8
27.	Zingiberenol	−7.7
28.	Trans -α-Bisabolene epoxide	−7.5
29.	Caryophylla-4(12),8(13)-dien-5-α-ol	−8.1
30.	Bicyclo [7.2.0]undecan-3-ol,11,11-dimethyl-4,8-bis(methylene)-	−8.2
31.	Caryophylla-4(12),8(13)-dien-5-β-ol	−7.2
32.	Myristic acid	−6.3
33.	Benzyl Benzoate	−8.3
34.	n-Cetyl alcohol	−6.1
35.	Palmitoleic acid	−6.9
36.	Isopropyl palmitate	−6.5
37.	cis-13-Octadecenoic acid	−6.2
38.	cis-Vaccenic acid	−6.7
39.	Stearic acid	−6.4
40.	Cannabichromene	−8.8
41.	Cannabicoumaronone	−8.1
42.	2-Methylnonacosane	−7.1

## Data Availability

The data are within the manuscript.

## Supplementary Materials

The following supporting information can be downloaded at: https://www.mdpi.com/article/10.3390/ijms26178552/s1.

## Abbreviations

The following abbreviations (the standard three-letter abbreviations) for the amino acids found in proteins, as well as other abbreviations, are used in this manuscript:

Ile	Isoleucine
Leu	Leucine
Phe	Phenylalanine
Arg	Arginine
His	Histidine
Ser	Serine
Trp	Tryptophan
Val	Valine
Tyr	Tyrosine
Lys	Lysine
Ala	Alanine
Thr	Threonine
Gly	Glycine
CAT	Catalase (enzyme)
GSH	Reduced Glutathione
IC_50_	Inhibitory concentration
ABS_Sample_	Absorbance value of samples
ABS_Control_	Absorbance value of control
DNA	Deoxyribonucleic Acid
NOX2	NADPH Oxidase 2
NADPH	Nicotinamide Adenine Dinucleotide Phosphate (Reduced form)
