# Supplementary material for: Chemical Composition and Antioxidant Activity of the Stembark Essential Oils of Two Cannabis sativa L. Cultivars from Komga, South Africa
Source: Int J Mol Sci. 2025 Sep 3;26(17):8552. doi: 10.3390/ijms26178552 (PMC12429610; doi:10.3390/ijms26178552)
Supplement: Supplementary file 1 [file ijms-26-08552-s001.zip › ijms-3749752-supplementary.pdf]

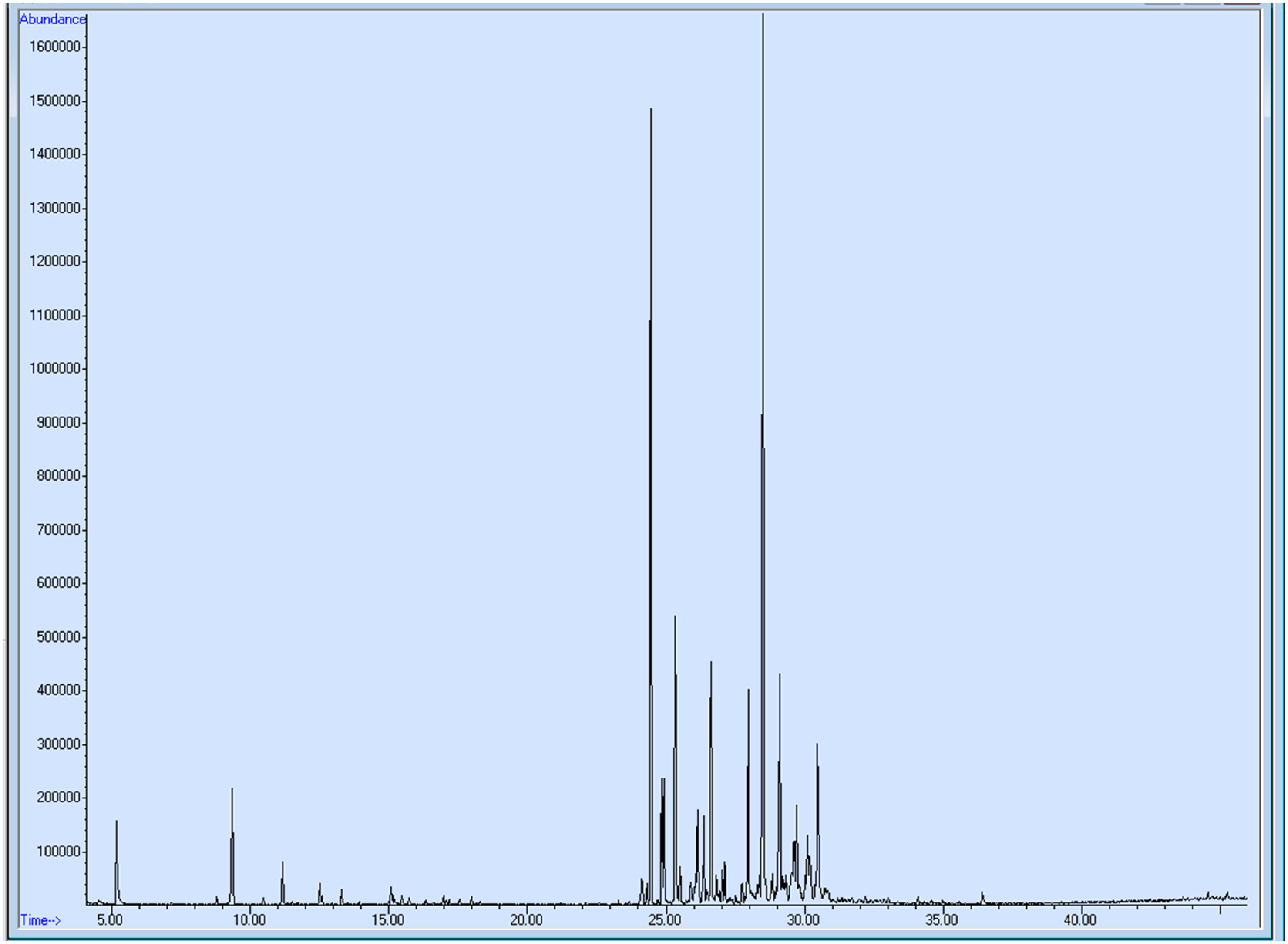

Figure S1: GC-MS Total Ion Chromatograms of fresh Lifter stembark oil (LSO) [RI (retention index) measured relative to n-alkanes (C9-C36) using ZB-5MS capillary column].

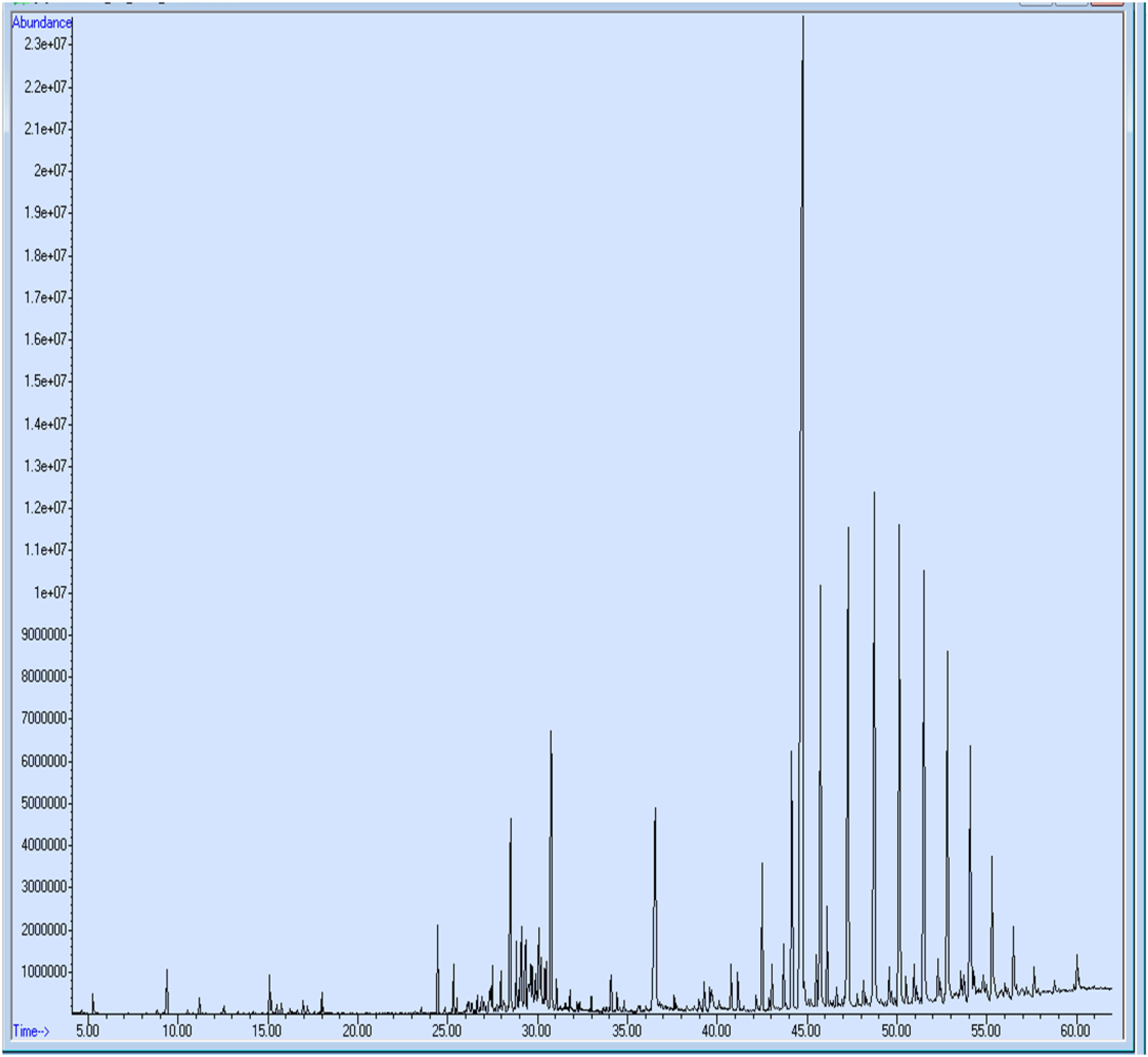

Figure S2: GC-MS Total Ion Chromatograms of dried Lifter stembark oil (DLSO) [RI (retention index) measured relative to n-alkanes (C9-C36) using ZB-5MS capillary column].

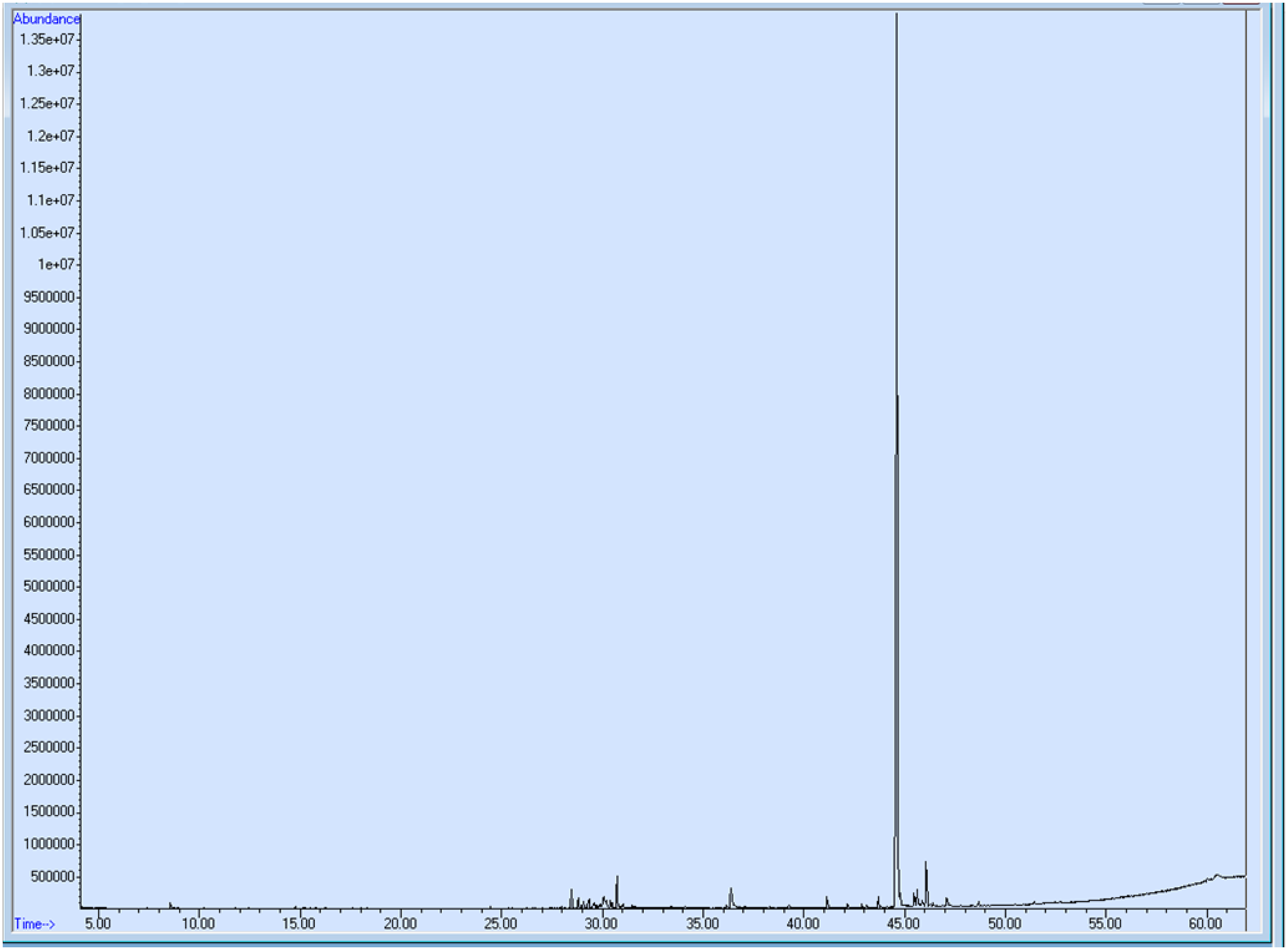

Figure S3: GC-MS Total Ion Chromatograms of fresh Cherrywine stembark oil (CSO) [RI (retention index) measured relative to n-alkanes (C9-C36) using ZB-5MS capillary column].

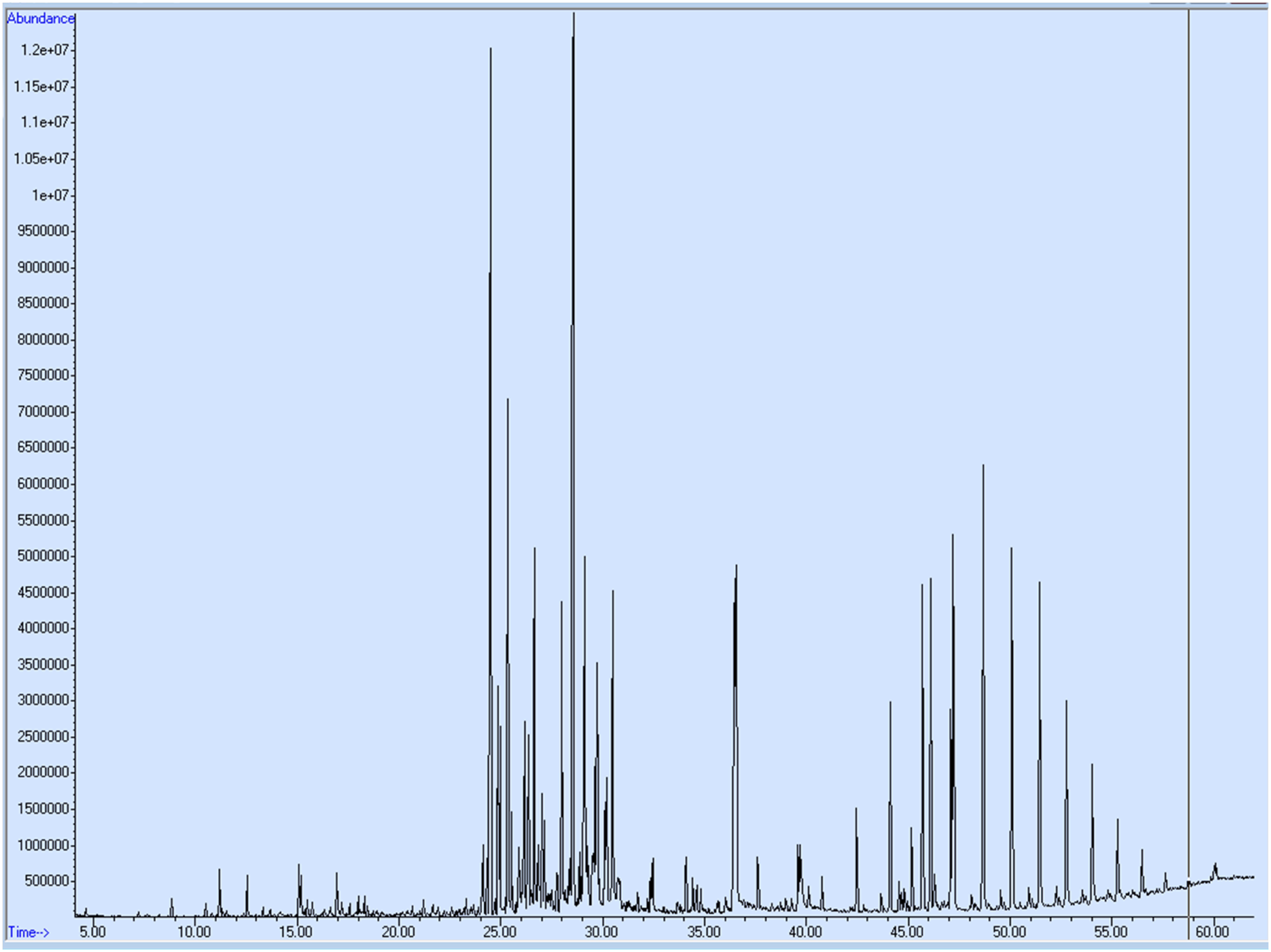

Figure S4: GC-MS Total Ion Chromatograms of dried Cherrywine stembark oil (DCSO) [RI (retention index) measured relative to n-alkanes (C9-C36) using ZB-5MS capillary column].
